# Chronic Wounds with Emphasis in Diabetic Foot Ulcers

**DOI:** 10.1155/2014/890352

**Published:** 2014-03-17

**Authors:** Jorge Berlanga-Acosta, David G. Armstrong, Gregory S. Schultz, Luis Herrera-Martinez

**Affiliations:** ^1^Tissue Repair and Cyto-Protection Research Laboratory, Center for Genetic Engineering and Biotechnology, P.O. Box 6162, Havana, Cuba; ^2^Southern Arizona Limb Salvage Alliance (SALSA), University of Arizona College of Medicine, AZ, USA; ^3^Department of Obstetrics and Gynecology, Institute for Wound Research, University of Florida, Gainesville, FL, USA; ^4^Center for Genetic Engineering and Biotechnology, P.O. Box 6162, 10600 Havana, Cuba

Since the seminal contribution of Frederick Banting and Charles Best, medicine, in the field of diabetes therapy was revolutionized. From that moment on, insulin therapy eliminated ketoacidosis as a principal cause of death among diabetics. As a result, diabetic patients enjoyed a longer lifespan and since then, novel pharmacological interventions have definitively contributed to their metabolic homeostasis. However, it is clear that traditional insulin therapy combined with emerging novel approaches did not translate into a significant reduction of major complications that lead to morbidity and mortality in patients with type II diabetes. Thus, these basic disease complications' as neuropathy, macro and microangiopathy, nephropathy, retinopathy and so many others; remain as challenge for scientists and clinicians. About 20 years ago an exciting concept emerged from large and well-controlled clinical trials. Perhaps the foremost message of these trials was the demonstration that once initiated; complications persisted and continued to progress, even in those subjects in which good metabolic control was achieved. It was the birth or the coining of the “Metabolic Memory” concept. To our understanding, only a deep and broad penetration in the pathophysiology of metabolic memory will help in the control, drugging and alleviation of diabetic complications.

Efforts and progresses have been achieved along the last few years. First, it is the elegant Michael Brownlee's unifying mechanistic hypothesis which in essence proposes that intracellular hyperglycemia causes increased mitochondrial reactive oxygen species production, which pulls the trigger to a vast number of downstream toxic events. Furthermore, this basic knowledge has expanded from a damaged and dysfunctional mitochondrial DNA, to the endoplasmic reticulum stress and the imprinting of a gone awry epigenetic control which seems to perpetuate the diabetic phenotype. At the end, all the roads converge to predispose diabetic cells to apoptosis, autophagy, early aging, etc. Diabetic foot ulceration is one of the most frightened diabetic complications, leading to disability, social exclusion and early mortality. The observation that diabetic patients exhibit a failure in their healing mechanism is ancestral. However, today this problem remains an unmet medical need, since diabetic population contribute to 80% of all non-traumatic lower extremities amputation around the world. Studies have shown that even in those patients with normal lower limb perfusion, good metabolic control, and a comprehensive wound care; the processes of wound granulation, contraction and re-epithelialization appear slower that in non-diabetic subjects. This situation is further complicated given the susceptibility of diabetic subjects to control peripheral soft tissues infections.

The challenging truth is that skin cells cannot hide out from the long arm of the biochemical diabetic milieu and an imprinting is left in fibroblasts, vascular cells and keratinocytes. These cells when harvested from diabetic foot wounds and transferred to proper culture conditions still exhibit an abnormal behavior and short replicative life span. In other words, the same replicative refractoriness remains in their “memory” as if they were still living within the diabetic ulcer environment. Molecular characterization of these cells has remarked precocious cells senescence, an abnormal susceptibility to apoptosis and a tendency to proliferative arrest as major pillars presiding wound chronification.

Multiple mechanisms may be involved in the genesis of the chronic phenotype and healing stubbornness in diabetes. Biomed Research International has gifted us a tremendous example of sensitivity and attachment to the daily medical needs. The Journal has dedicated this special issue to focus in the biology, and clinical and surgical aspects of chronic wounds with special emphasis in diabetic foot ulcers. The issue is varied and rich by including a series of basic and clinical studies, as well as reviews that will certainly furnish and satisfy a broad audience of readers. This number is therefore an additional and significant contribution in the struggle against a mutilating diabetic complication.

*Jorge Berlanga-Acosta* *David G. Armstrong* *Gregory S. Schultz* *Luis Herrera-Martinez*

